# Patient Perceptions of In Vivo Versus Virtual Reality Exposures for the Treatment of Anxiety Disorders: Cross-Sectional Survey Study

**DOI:** 10.2196/47443

**Published:** 2023-10-16

**Authors:** Amanda N Levy, Vasileios Nittas, Tyler B Wray

**Affiliations:** 1 Department of Computer Science Brown University Providence, RI United States; 2 Department of Behavioral and Social Sciences Brown University Providence, RI United States

**Keywords:** counseling treatment, phobias, PTSD, patient perspective, in vivo exposures, virtual reality, exposure therapy, anxiety, psychotherapy, effectiveness, digital therapy, affective disorders

## Abstract

**Background:**

Psychotherapy, and particularly exposure therapy, has been proven to be an effective treatment for many anxiety disorders, including social and specific phobias, as well as posttraumatic stress disorders. Currently, exposures are underused and mostly delivered in vivo. Virtual reality exposure therapy (VRET) offers a more flexible delivery mechanism that has the potential to address some of the implementation barriers of in vivo exposures while retaining effectiveness. Yet, there is little evidence on how patients perceive different exposure therapy methods.

**Objective:**

This study aims to explore the perceptions of individuals with anxiety disorders toward in vivo and VRET. Our findings can inform therapists about the degree of patient interest in both methods while exploring the demand for VRET as an alternative and novel treatment approach.

**Methods:**

Web-based survey assessing the (1) interest in, (2) willingness to use, (3) comfort with, (4) enthusiasm toward, and (5) perceived effectiveness of exposure therapy when delivered in vivo and through VR. Participants included individuals with specific phobia, social phobia, posttraumatic stress disorder, or acute stress disorder or reaction. Participants were presented with educational videos about in vivo and VRET and asked to provide their perceptions quantitatively and qualitatively through a rated scale and free-text responses.

**Results:**

In total, 184 surveys were completed and analyzed, in which 82% (n=151) of participants reported being willing to receive in vivo exposures and 90.2% (n=166) reported willingness to receive VRET. Participants reported higher interest in, comfort with, enthusiasm toward, and perceived effectiveness of VRET compared to in vivo. Most reported in vivo concerns were linked to (1) increased anxiety, (2) feelings of embarrassment or shame, and (3) exacerbation of current condition. Most reported VRET concerns were linked to (1) risk of side effects including increased anxiety, (2) efficacy uncertainty, and (3) health insurance coverage. The most frequently mentioned VRET benefits include (1) privacy, (2) safety, (3) the ability to control exposures, (4) comfort, (5) the absence of real-life consequences, (6) effectiveness, and (7) customizability to a wider variety of exposures.

**Conclusions:**

On average, our participants expressed positive perceptions toward exposure therapy, with slightly more positive perceptions of VRET over in vivo exposures. Despite valid personal concerns and some misconceptions, our findings emphasize that VRET provides an opportunity to get much-needed therapy to patients in ways that are more acceptable and less concerning.

## Introduction

About 1 in 5 American adults experience an anxiety disorder, such as social anxiety disorder, specific phobias, or generalized anxiety disorder, making it the most common mental health condition in the United States [1]. Anxiety disorders cause a significant personal and public health burden, often leading to various degrees of disability, increased risk for comorbidities (eg, cardiovascular diseases), and premature mortality [2-4]. The economic burden of anxiety disorders is also high, with estimated annual direct health care costs exceeding US $34 billion [5]. Identifying novel, cost-effective ways to improve their treatment and management is therefore essential.

Both medications and psychotherapy are effective in treating anxiety disorders. Meta-analyses suggest that, for some conditions, psychotherapy may be more effective than medication alone, and the effects of psychotherapy may be more durable [6]. Almost all effective psychotherapy approaches for anxiety disorders incorporate some exposure techniques [7], and these exposure techniques likely have the strongest effects on patient outcomes [8,9]. Exposure techniques guide individuals to gradually interact with fear-provoking stimuli to learn safety mechanisms that counteract their anxiety-inducing perceptions of triggering situations [10]. In psychotherapy, exposure techniques are typically delivered in vivo, meaning that, counselors create tangible experiences surrounding the feared stimuli that patients physically confront in person [10,11].

Despite the power of these techniques, past research consistently shows that psychotherapists underuse exposures. A study of 684 psychotherapists revealed that therapists incorporated exposure therapy in only 46.8% (n=86) of their anxiety disorder treatments [12]. Negative beliefs (eg, low tolerability and low confidentiality), as well as logistical barriers (eg, required time and effort), may be some of the reasons therapists do not fully use exposures [12-14]. Using virtual reality (VR) to deliver exposure techniques may be one way to overcome some of these barriers.

VR is a computer-generated experience that provides an immersive illusion of reality by mimicking the way humans naturally perceive the world [15]. When effective, this illusion often evokes a sense of presence in users or a subjective feeling that they are physically present in the digital scenario [16]. VR-reality exposure therapy (VRET) involves using an immersive digital environment to deliver exposure techniques in a similar way to in vivo exposures, but using digital experiences. For specific phobias, posttraumatic stress disorder (PTSD), and social phobia, meta-analyses have found that VRET is as or similarly effective as in vivo exposure therapies [17-19]. VRET may also require less time, effort, and cost to deliver, while allowing therapists to personalize exposures without risks to patients’ confidentiality [20]. Together, these factors could suggest that VRET may be easier for therapists to adopt and use than in vivo exposures and ultimately lead to greater use of exposure techniques in therapy. However, several important therapist and patient barriers have also limited the use of VRET in practice to date, including lack of familiarity with the technology, the need for additional training, cost, and potential side effects among users [21-24] (eg, simulator sickness, a temporary condition similar to motion sickness that affects about 0.4% of VR users [25]). Another key barrier has been concerns among therapists and other stakeholders that patients may be reluctant or refuse to use VR in treatment [24-26]. Yet, an underexplored benefit of VRET is that it may actually be more acceptable to patients than in vivo exposures. If so, therapists may be more likely to retain patients in therapy using VR, and providing therapists with evidence of its acceptability among patients could help encourage more use of VRET among therapists. Few studies to date have surveyed and compared perceptions of effectiveness, interest, comfort, and enthusiasm about VRET versus in vivo exposures among patients.

In this study, we surveyed individuals experiencing from anxiety disorders about their perceptions of in vivo and VR exposure therapy. We also explored whether various aspects of patients’ treatment history and mental health status were associated with greater interest in VRET relative to in vivo exposure. Our findings aim to inform therapists about the degree of patient interest in both methods while exploring the demand for VRET as an alternative and novel treatment approach. Finally, our results could inform and guide implementation strategies that target VRET adoption.

## Methods

### Overview

We conducted a web-based survey assessing the (1) interest in, (2) willingness to use, (3) comfort with, (4) enthusiasm toward, and (5) perceived effectiveness of exposure therapy, in vivo and through VR. We selected these measurement constructs because they are indicators of patient demand and acceptance of exposure therapy as a treatment. Participants included individuals with specific phobia, social phobia, PTSD, or acute stress disorder or reaction. The survey asked participants for basic information about their anxiety disorder and treatment history, presented educational videos about in vivo and VRET, and recorded participant opinions of each exposure approach. Participants shared their perceptions quantitatively and qualitatively through a rated scale and free-text responses.

### Participants

Advertisement and recruitment were conducted on the web, via Reddit posts and advertisements on Facebook and Instagram, as well as through fliers, mailed to regional wellness centers. Through a QR code (flyers) or a link (web-based advertisement), potential participants were directed to a landing page followed by a brief survey that assessed eligibility criteria. Participants were initially eligible if all of the following conditions were met: (1) 18 years or older, (2) able to speak and read English fluently, (3) diagnosed with a specific phobia, social phobia, posttraumatic stress disorder, or acute stress disorder or reaction by a person licensed to provide professional counseling for mental health conditions by a recognized licensing body in the United States, (4) experience frequent anxiety (in the past week, felt anxious “most of the time”), and (5) have a primary residence in the US states of Rhode Island, Massachusetts, or Connecticut. All participants were authenticated using reCAPTCHA 2.0 and 3.0 and by requiring agreement across repeated questions. We also matched participants’ self-reported state of residence with their geolocated information processing to confirm that they met the geographic eligibility. Before collecting personally identifying data and being referred to the main survey, those initially eligible were asked to provide their contact information and informed consent.

### Procedures

The main survey consisted of 6 parts. The first assessed basic information about participants’ mental health and treatment history, followed by a second part consisting of a 5-minute educational video about in vivo exposures. Video was created internally by our research team to provide a brief audio-visual overview of the treatment approach. A definition, example case, and efficacy statement were also included. The video page was locked for the video’s complete length to ensure that participants could not continue the survey until the time elapsed. There were 2 true or false questions that assessed whether participants had reviewed the content. Participants who answered at least one question incorrectly 2 or more times were not allowed to continue and were not included in our analyses. Those who passed the quiz were transferred to the survey’s third part, which assessed their perceptions of in vivo exposure therapy. The survey then transitioned to the fourth part, which involved showing participants a video introducing VR technology and its use in providing exposure to psychotherapy. This video was also locked until the video’s time elapsed, and 5 true or false follow-up questions assessed participants’ understanding. Those who answered all questions correctly passed the quiz and were transferred to the survey’s fifth part, which assessed their perceptions of VRET. The survey’s sixth part concluded with free-response questions on participants’ concerns toward, perceived benefits of, and general comments about VRET. The survey lasted between 30 and 45 minutes.

### Ethical Considerations

The Brown University Institutional Review Board approved all procedures (protocol 2022003298). All participants in this study provided informed consent prior to completing the survey. Eligible and interested participants were first provided basic information about the study in bulleted format, before being referred to the full, approved study consent document. Participants were allowed to take as much time as needed to consider this information and could view and print a copy of the full consent form. Participants then indicated their informed consent by clicking a radio button. Those who provided consent were then referred to the main survey. Participants’ confidentiality was protected by using a survey platform (Qualtrics) that required Brown credentials and 2-step validation. Identifying information about participants was also collected in a separate survey from their responses to key items and linked via an auto-generated, study-assigned, alphanumeric ID number. The link between these IDs and participants’ identifying information was then destroyed after all study data had been collected. Participants who completed the survey were provided with a US $15 Amazon gift card.

### Measures

#### Demographic Characteristics

We gathered information on participants’ age, gender, relationship status, ethnicity, race, education, total annual individual income, employment status, and sexual identity.

#### Mental Health and Treatment History

We asked participants about their anxiety disorder diagnoses (specific phobia, social phobia, PTSD, or acute stress disorder or reaction), the date of their original diagnosis, and their treatment history. Items assessed which types of treatments they had received (counseling, prescription medication, and procedures [eg, Transcranial Magnetic Stimulation and Eye Movement Desensitization and Reprocessing]), the length of any treatments (if applicable), and reasons for not receiving treatment (eg, fear of worsening anxiety and ashamed for needing help).

#### Severity of Anxiety Symptoms

We used the Overall Anxiety Severity and Impairment Scale (OASIS) [27] to assess the current severity of participants’ anxiety symptoms. The OASIS uses a 0-4 (0 indicating None and 4 indicating Extreme) rating scale on 4 questions about the severity of their anxiety and impairment.

#### Perceptions of In Vivo and VR Exposures

To assess participants’ perceptions of both in vivo and VRET, we asked participants to rate their willingness to use, comfort with, interest in, and enthusiasm toward the exposure approach, as well as their perceptions about the effectiveness of each approach. Participants rated each item on a 1 (not at all effective/interested/comfortable/enthusiastic) to 4 (very or extremely effective/interested/comfortable /enthusiastic) scale.

### Data Analysis

We computed basic descriptive and summary statistics of participants’ demographic and mental health characteristics (mean, SD, and percentage). To compare participants’ perceptions of effectiveness, interest, comfort, and enthusiasm toward in vivo and VR exposure therapy, we estimated paired *t* tests. To test whether aspects of participants’ mental health and treatment history were associated with differential perceptions of VR exposures compared to in vivo, we computed an outcome variable reflecting participants’ ratings of their interest in VR exposures and subtracted their ratings of interest in in vivo exposures. We then estimated a linear regression model with this variable as the primary outcome, additionally including participants’ diagnosed conditions, time since diagnosis, the types and number of previous treatments, treatment length, and total, standardized OASIS scores. We arrived at a final, parsimonious model using a “tear down” approach in which nonsignificant variables (*P*>.20) were deleted from an initial model that included all covariates. Finally, we categorically reviewed participants’ responses to free-text-entry questions about the concerns and perceived benefits of VRET for common themes and calculated the frequency of each theme.

## Results

### Overview

In total, 1698 started the screening, and of these, 184 fulfilled all eligibility criteria, provided informed consent, and were included in our analysis. Participants were primarily young adult females, largely non-Hispanic White. More than half were college-educated, employed, and with a reported income of above US $50,000. Table 1 provides all participant characteristics.

**Table 1:** Participant characteristics.

Characteristic		Values
Age in years, mean (SD)		29.95 (10.79)
**Current gender, n (%)**		
	Man	43 (23.4)
	Woman	105 (57.1)
	Trans or other	36 (19.6)
Relationship, not committed, n (%)		101 (55)
**Ethnicity, n (%)**		
	Hispanic or Latino	19 (10.3)
	Not Hispanic or Latino	165 (89.7)
**Race, n (%)**		
	American Indian or Alaska Native	1 (0.54)
	Asian	3 (1.6)
	Black or African-American	57 (31)
	Multiracial	6 (3.3)
	White	113 (61.4)
	Chose not to respond	4 (2.2)
**Education, n (%)**		
	College or higher degree	98 (53.3)
	Lower than a college degree	86 (46.7)
Income, above US $30,000, n (%)		105 (57.1)
Employment, currently employed, n (%)		97 (52.7)
**Sexual orientation or identity, n (%)**		
	Heterosexual	54 (29.4)
	Gay or lesbian	56 (30.4)
	Bisexual	53 (28.8)
	Other or not sure	21 (11.4)

In total, 56% (103/184) of participants reported having more than 1 diagnosed anxiety disorder. Specific phobia was the most reported condition (110/184, 59.8%), followed by PTSD (96/184, 52.2%). On average, the mean time since diagnosis was about 2 (SD 1.79) years. Participants reported experiencing symptoms of anxiety frequently (mean 3.13, SD 0.52) with moderate to severe intensity (mean 2.72, SD 0.70). About 77% (n=142) of participants had, at some point, received treatment for their anxiety disorders, with 96% (n=136) of those reporting counseling, 65% (n=92) medication intake, and 7% (n=10) other procedures (eg, biofeedback, eye movement desensitization and reprocessing, and transcranial magnetic stimulation). Those who had never received treatment (42/184, 23%), most commonly reported not believing in its effectiveness, followed by feelings of shame and concerns that their anxiety could worsen.

### In Vivo Versus VR Exposure Therapy

In total, 61% (n=112) of all participants had previously received exposure therapy. Participants reported a mean average of 13.13 (SD 13.79) hours of in vivo exposure therapy and 10.53 (SD 13.79) hours of imaginal exposure therapy. In total, 7% (n=13) of all participants had previously done VR exposure therapy. All participants ranked on a scale of 1 to 4 (1 indicating none and 4 indicating extreme) their concerns about engaging in in vivo exposures. The top three concerns were (1) increased level of anxiety during an exposure (mean 3.03, SD 0.88), (2) feelings of embarrassment or shame when being seen in public during an exposure (mean 2.64, SD 1.06), and (3) doubts that it would improve anxiety as well as concerns that it might exacerbate it (mean 2.63, SD 1.04).

Despite these concerns, 82% (n=151) of participants reported being willing to receive in vivo and 90.2% (n=166) of participants were willing to receive VRET. On average, participants reported higher perceived effectiveness for VRET compared to in vivo exposures. Similarly, participants reported higher interest in VRET than in vivo exposures. Comfort was also ranked higher for VRET compared to in vivo exposures, while participants were on average more enthusiastic toward VRET than toward in vivo exposures. All differences were statistically significant. See Table 2 for descriptive statistics and *t* tests*.*

**Table 2:** Comparing patient perceptions of in vivo versus virtual reality exposure therapy^a^.

	In vivo, mean (SD)	Virtual reality, mean (SD)	*t* test (*df*)	*P* value
Perceptions of effectiveness	2.96 (0.05)	3.15 (0.06)	–3.27 (178)	.001
Interest	3.06 (0.06)	3.33 (0.06)	–4.46 (178)	<.001
Comfort	2.78 (0.07)	3.25 (0.06)	–6.65 (178)	<.001
Enthusiasm	2.65 (0.07)	3.16 (0.06)	–7.46 (178)	<.001

^a^Values reported from a paired samples *t* test.

### Perceived Benefits of VR Exposure Therapy

Perceived VRET benefits were frequency-coded from free-text responses. The most frequently mentioned benefits include privacy, safety, the ability to control exposures, comfort, the absence of real-life consequences, effectiveness, and customizability to a wider variety of exposures. Table 3 provides all perceived benefits in detail.

**Table 3:** Perceived benefits of VRET^a^.

Perceived benefits	Values, n (%)
VRET^a^ can be done in private	21 (11.4)
VRET feels like a safe, controlled, and comfortable experience	20 (10.9)
VRET is detached from real-life consequences	12 (6.5)
VRET is effective	11 (6.0)
VRET is customizable for a wider variety of exposures	9 (4.9)
VRET is easy-to-use, accessible	7 (3.8)
VRET is fun and enjoyable	7 (3.8)
VRET is a drug-free method	4 (2.2)
VRET is accessible for those with disability	2 (1.1)

^a^VRET: virtual reality exposure therapy.

### Concerns About VR Exposure Therapy

Reported concerns about VRET were also extracted from free-text responses. Frequency coding revealed most participants were concerned about potential side effects, treatment efficacy, and costs. Participants’ specific concerns related to side effects were eye or vision issues, migraines, and motion sickness. Participants were also concerned that VRET might not be efficacious or applicable to certain anxieties (eg, domestic and sexual abuse), might increase anxiety and discomfort, and is not based on enough evidence. Concerns about cost seemed to relate more to general concerns about health insurance coverage, instead of VR itself. Table 4 provides all reported concerns in more detail.

**Table 4:** Reported concerns of VRET^a^.

Concern	Values, n (%)
VRET^a^ may cause side effects	36 (19.6)
VRET is not efficacious	31 (16.8)
VRET is not based on enough evidence yet	23 (9.2)
VRET is costly and might not be covered by health insurance	22 (12)
VRET might increase anxiety and discomfort	18 (9.8)
VRET might not help with events related to anxiety, such as childhood trauma or domestic abuse	15 (8.2)
VRET is not easily accessible	9 (4.9)
VRET takes time to be effective	6 (3.3)

^a^VRET: virtual reality exposure therapy.

### Factors Associated With Differential Interest in VRET Versus In Vivo Exposures

Finally, in linear regression models testing factors associated with a preference for VRET over in vivo exposures, those who reported having been diagnosed with more anxiety conditions were more likely to report more interest in VRET relative to in vivo. Similarly, those who had been struggling with their conditions for longer preferred VRET. Finally, those who had completed more sessions of psychotherapy reported less differential interest in VRET relative to in vivo exposures. See Table 5 for results.

**Table 5:** Linear regression of differential interest in VRET^a^ relative to in vivo exposures.

Variable	β	SE	*P* value	95% CI
Number of anxiety conditions	.30	0.11	.008	0.08 to 0.52
Time since diagnosed	.13	0.06	.026	0.02 to 0.24
OASIS^b^ total score	–.15	0.10	.153	–0.35 to 0.05
Number of psychotherapy sessions, lifetime	–.12	0.06	.043	–0.24 to –0.01

^a^VRET: virtual reality exposure therapy.

^b^OASIS: Overall Anxiety Severity and Impairment Scale.

## Discussion

### Principal Findings

Our findings suggest that while adults with certain anxiety disorders often prefer VRET to in vivo exposures, participants rated both approaches very positively. Participants were generally more interested in, comfortable with, and enthusiastic toward VRET over in vivo. Perceived effectiveness was also higher on average for VRET, despite noting equivalent effectiveness in our informational videos, although only 8% (n=15) reported perceiving that VRET was more effective than in vivo. Given that only 61% (n=112) of participants had experienced any form of exposure therapy before, VR could present an opportunity to provide effective psychotherapy for anxiety disorders in a more palatable form, particularly for those who are newer to psychotherapy. This possibility is further supported by the results of our regression model, which showed that those with less experience with psychotherapy generally reported stronger interest in VRET relative to in vivo exposures. Similarly, findings from our regression model showing that participants with greater comorbidities and who had been struggling with their anxiety conditions for longer had a stronger interest in VRET relative to in vivo exposures could suggest that VRET could also be a good fit for those who may have found relief elusive or who are contending with many conditions for which VRET might be helpful.

Our findings also suggest that VRET may help address key concerns patients have about in vivo exposures. The risk of experiencing severe anxiety, embarrassment, or shame when completing exposures in public was among the top concerns participants reported for in vivo exposures, which VRET may address by enabling therapists to conduct exposures in the privacy of their offices. The inability to control the intensity of exposure was also a common concern, which VRET could reduce by allowing therapists to more carefully control the pace and severity of exposures. These advantages are consistent with participants’ ratings of the top benefits of VRET, which were its privacy and control.

Participants also had concerns about VRET, however. Among the most common were worries about potential side effects of VR, such as feeling nauseated, dizzy, or getting a headache. These concerns are certainly valid since it is well-known that some VR users can experience “simulator sickness,” a condition similar to motion sickness [28]. However, modern VR systems incorporate a number of ergonomic principles that have been shown to reduce the risk of simulator sickness, such as motion blurring and faster refresh rates [29,30]. Studies with these more modern VR systems have shown that approximately 0.4% of participants report symptoms of simulator sickness [25]. Symptoms are also typically mild, reverse soon after discontinuation, and occur least often among adults [31]. Participants were also concerned about the efficacy of VRET, despite our educational videos explicitly noting their comparable efficacy relative to in vivo exposures. However, like similar reservations among therapists, this concern might be addressed with more explicit, dedicated efforts to disseminate the findings of existing research, which strongly supports the efficacy of VRET [17-19]. Finally, concerns about the cost and insurance coverage of VRET are reasonable, given that no clear payment model has yet been established for VRET in part due to its limited uptake among therapists. However, the handful of therapists who have adopted VRET to date most often bill for VR-assisted services in the same way as more typical therapy services, resulting in no additional costs to patients. If adoption increases in the future, clearer and more reliable payment models are likely to be established [31-34].

### Limitations and Future Research

A number of important limitations in this study are important to note. First, these findings are based on a relatively small sample of participants who completed a web-based survey. Findings may vary in larger samples or in studies using different methods. Our sample was also limited to individuals residing in the US states of Rhode Island, Massachusetts, and Connecticut, and so, these findings may not generalize to those in other geographic areas. Next, our survey relied entirely on participants’ self-report about their anxiety disorder diagnoses. Research using more careful methods to assess specific diagnoses, such as those using clinical interviews, may yield different results. Additionally, a relatively high percentage of participants (112/184, 61%) had previously completed at least some exposure therapy, whereas a low percentage of participants had tried VRET. Thus, there might be bias when comparing reactions to traditional in vivo exposures and VRET. Moreover, given the structure of the survey, patients always answered questions about in vivo exposures first, before answering questions about VRET. This ordering may have led participants to respond differently than if the order were reversed. Last, we were required to specify the study’s topic in all recruitment materials. Although this brief description did not emphasize technology, our recruitment materials may have attracted participants who were particularly motivated to participate in research on anxiety disorder treatment. Thus, results may be different among participants with less interest in this topic.

This research also represents an early first step toward understanding patient factors in the uptake of VRET. Future research should explore these factors in more realistic populations and settings, such as among patients presenting to mental health clinics. Future research could also begin exploring specific strategies for encouraging further uptake of VR among patients.

### Conclusions

On average, our participants expressed positive perceptions toward exposure therapy. However, they indicated higher levels of willingness, interest, comfort, enthusiasm, and perceived effectiveness for VRET than for traditional in vivo exposures. The perceived benefits of VR, which include higher privacy, controllability, safety, and comfort may also mitigate some of the major concerns participants had toward in vivo exposure therapies. As such, VRET may be a more palatable alternative to traditional in vivo exposures. Future research should focus on designing and testing explicit strategies to improve VRET adoption in community mental health treatment settings.

## Abbreviations

OASIS: Overall Anxiety Severity and Impairment Scale
PTSD: posttraumatic stress disorder
VR: virtual reality
VRET: virtual reality exposure therapy

## Data Availability

The data are not publicly available but can be provided upon request to the authors.
